# Supplementary material for: Views of healthcare professionals regarding barriers and facilitators for a Fracture Liaison Service in Malaysia
Source: PLoS One. 2024 Jul 26;19(7):e0307919. doi: 10.1371/journal.pone.0307919 (PMC11280531; doi:10.1371/journal.pone.0307919)
Supplement: S1 Appendix — (PDF) [file pone.0307919.s001.pdf]

S1 Appendix. Full topic guide for different stakeholders.

Interview Topic Guide: Doctors

1. Do you manage patients with fractures? Can you elaborate on your involvement?
2. How do you usually treat patients with fractures? (Probe further - Is there any difference between how you would treat patients with fragility fractures versus other fractures?)
3. In your opinion, how is the delivery of secondary fracture prevention in the University Malaya Medical Centre currently? Can you elaborate further?
4. Have you ever heard of the term Fracture Liaison Service (alternative: secondary fracture prevention service)? If yes, can you explain a bit further; If no, can you try to guess? (Provide definition: coordinator-based, secondary fracture prevention services implemented by health care systems for the treatment of osteoporosis patients) (Secondary fracture prevention: Early identification of the first fracture and prevent future fracture)
5. Have you participated in any similar service previously? If yes, can you tell us more about the service?
6. Are you aware of any other hospitals in Malaysia that have a Fracture Liaison Service? If yes, can you describe that service?
7. What do you think should be the functions of a Fracture Liaison Service? (eg. coordinate care between different disciplines, identify fragility fracture patients, referral for bone health assessment and investigations, ensure treatment initiation and adherence)
8. What are the possible benefits of implementing a Fracture Liaison Service in the University Malaya Medical Centre? (eg. treatment initiation and adherence, re-fracture rates and mortality, bone health assessments, and coordination between departments)
9. In your opinion, is there a need for a Fracture Liaison Service in the University Malaya Medical Centre? Why?
10. What are the difficulties that might be faced when implementing the Fracture Liaison Service in the University Malaya Medical Centre? (eg. financial, manpower, equipment, resources, patients' willingness)
11. Who should be involved in the Fracture Liaison Service? Why?
12. In your opinion, is a Fracture Liaison Service coordinator required to ensure the success of this service? Why?
13. Which healthcare professional can serve as a Fracture Liaison Service coordinator? Why?
14. What factors do you think would facilitate the establishment of a Fracture Liaison Service in Malaysia? (eg. guidelines, organisation support)
15. Do you have anything else to add regarding Fracture Liaison Service?

Interview Topic Guide: Pharmacists

1. What do you think about the importance of secondary fracture prevention? (Secondary fracture prevention: Early identification of the first fracture and prevent future fracture)
2. In your opinion, how is the delivery of secondary fracture prevention in the University Malaya Medical Centre currently? Why?
3. Have you ever heard of the term Fracture Liaison Service (alternative: secondary fracture prevention service)? If yes, can you explain a bit further; If no, can you try to guess? (Provide definition: coordinator-based, secondary fracture prevention services implemented by health care systems for the treatment of osteoporosis patients)
4. Have you participated in any similar service previously? If yes, can you tell us more about the service?
5. Are you aware of any other hospitals in Malaysia that have a Fracture Liaison Service? If yes, can you describe that service?
6. What do you think should be the functions of a Fracture Liaison Service? (eg. coordinate care between different disciplines, identify fragility fracture patients, referral for bone health assessment and investigations, ensure treatment initiation and adherence)
7. What are the possible benefits of implementing a Fracture Liaison Service in the University Malaya Medical Centre? (eg. treatment initiation and adherence, re-fracture rates and mortality, bone health assessments, and coordination between departments)
8. In your opinion, is there a need for a Fracture Liaison Service in the University Malaya Medical Centre? Why?
9. What are the difficulties that might be faced when implementing the Fracture Liaison Service in the University Malaya Medical Centre? (eg. financial, manpower, resources)
10. Who should be involved in the Fracture Liaison Service?
11. How do you think pharmacists can contribute to a Fracture Liaison Service? If yes, please elaborate further (probe – coordinator, counselling, public health, might ask what are the barriers to these)
12. In your opinion, is a Fracture Liaison Service coordinator required to ensure the success of this service? Why?
13. Which healthcare professional (pharmacist) can serve as a Fracture Liaison Service coordinator? Why?
14. What factors do you think would facilitate the establishment of a Fracture Liaison Service in Malaysia? (eg. guidelines, organisation support)
15. Do you have anything else to add regarding Fracture Liaison Service?

Interview Topic Guide: Nurses

1. How are you involved in the care of fracture patients?
2. What do you think about the importance of secondary fracture prevention? (Secondary fracture prevention: Early identification of the first fracture and prevent future fracture)
3. In your opinion, how is the delivery of secondary fracture prevention in the University Malaya Medical Centre currently? Can you elaborate further?
4. Have you ever heard of the term Fracture Liaison Service (alternative: secondary fracture prevention service)? If yes, can you explain a bit further; If no, can you try to guess? (Provide definition: coordinator-based, secondary fracture prevention services implemented by health care systems for the treatment of osteoporosis patients)
5. Have you participated in any similar service previously? If yes, can you tell us more about the service? (eg. nurse coordinator for diabetes)
6. Are you aware of any other hospitals in Malaysia that have a Fracture Liaison Service? If yes, can you describe that service?
7. What do you think should be the functions of a Fracture Liaison Service? (eg. coordinate care between different disciplines, identify fragility fracture patients, referral for bone health assessment and investigations, ensure treatment initiation and adherence)
8. What are the possible benefits of implementing a Fracture Liaison Service in the University Malaya Medical Centre? (eg. treatment initiation and adherence, re-fracture rates and mortality, bone health assessments, and coordination between departments)
9. In your opinion, is there a need for a Fracture Liaison Service in the University Malaya Medical Centre? Why?
10. What are the difficulties that might be faced when implementing the Fracture Liaison Service in the University Malaya Medical Centre? (eg. financial, manpower, resources)
11. Who should be involved in the Fracture Liaison Service?
12. How do you think nurses can contribute to a Fracture Liaison Service? If yes, please elaborate further (probe – coordinator, patient education, might ask what are the barriers to these)
13. In your opinion, is a Fracture Liaison Service coordinator required to ensure the success of this service? Why?
14. Which healthcare professional (nurse) can serve as a Fracture Liaison Service coordinator? Why?
15. What factors do you think would facilitate the establishment of a Fracture Liaison Service in Malaysia? (eg. guidelines, organisation support)
16. Do you have anything else to add regarding Fracture Liaison Service?

Interview Topic Guide: Policymakers

1. What do you think about the current fracture management at University Malaya Medical Centre? [eg. Is there any area that is lacking or needs improvement? or might probe - how is the delivery of secondary fracture prevention at University Malaya Medical Centre currently? Can you elaborate further? (Secondary fracture prevention: Early identification of the first fracture and prevent future fracture)]
2. Have you ever heard of the term Fracture Liaison Service (alternative: secondary fracture prevention service)? If yes, can you explain a bit further; If no, can you try to guess? (Provide definition: coordinator-based, secondary fracture prevention services implemented by health care systems for the treatment of osteoporosis patients)
3. Are you aware of any other hospitals in Malaysia that have a Fracture Liaison Service? If yes, can you describe that service?
4. What do you think should be the functions of a Fracture Liaison Service? (eg. coordinate care between different disciplines, identify fragility fracture patients, referral for bone health assessment and investigations, ensure treatment initiation and adherence)
5. What are the possible benefits of implementing a Fracture Liaison Service in the University Malaya Medical Centre? (eg. treatment initiation and adherence, re-fracture rates and mortality, bone health assessments, and coordination between departments)
6. In your opinion, is there a need for a Fracture Liaison Service at the University Malaya Medical Centre? Why?
7. As a policymaker, what is the most important consideration in implementing a new service in the University Malaya Medical Centre?
8. What other aspects of the service would influence your decision-making process of a new service implementation? (If they are aware of FLS, can probe further into anything specific to FLS) (eg. guidelines, organisation support, the personnel behind it, competition, fame, novelty)
9. What are the difficulties that might be faced when implementing the Fracture Liaison Service in the University Malaya Medical Centre? (eg. financial, manpower, equipment, resources)
10. Who should be involved in the Fracture Liaison Service?
11. Do you have anything else to add regarding Fracture Liaison Service?
