# Supplementary material for: Views of healthcare professionals regarding barriers and facilitators for a Fracture Liaison Service in Malaysia
Source: PLoS One. 2024 Jul 26;19(7):e0307919. doi: 10.1371/journal.pone.0307919 (PMC11280531; doi:10.1371/journal.pone.0307919)
Supplement: S2 Appendix — (PDF) [file pone.0307919.s002.pdf]

S2 Appendix. Illustrative quotations of the study themes.

Theme 1: Current delivery of secondary fracture prevention

Codes	Subcodes	Key points	Quotations
Current delivery of secondary fracture prevention	Positive perceptions	• Many patients were started on osteoporosis treatment	• … I think is ok, because … most of the doctors will start the patient on calcium and vitamin D, that is what I can see, and for me working in the [pharmacy] store also the order for calcium and vitamin D is a lot.
			• I think that our hospital, they are doing well. Their treatment, their guidelines are very well done.
			• Ya, yes, I do feel that a lot of patients have been given the right care, the right medication and the right suitability of the medication to prevent their bone loss, perhaps, from what I have seen a lot of them were given like vitamin D, supplement … calcium, perhaps some of them be needing like injection like Zometa … to prevent even more at the higher stage I guess, so I think yes, we are having a good … kind of like… delivery care in the … sense of medication.
			• … because we are working in a pharmacy, I am a pharmacist by profession, so we see a lot of osteoporosis clinic family physicians prescribing rocaltrol at the same time calcium supplement for the patient as a health supplement to strengthen the bone to prevent osteoporosis. At the same time, we also see … doctors prescribing bisphosphonate for osteoporosis treatment. So I think at our hospital, we are doing good in treating osteoporosis and managing patients with osteoporosis … diseases.
			• If a patient has a femur fracture, we will give him some advice on the importance (of fall prevention) prior to discharge, we advise him to take care, … for someone to accompany them to the toilet … wear non-slip shoes...
			• Yeah, is acute management because I'm an emergency physician, and I do see everyday fractures in emergencies. So, I can say that our fracture management in emergency medicines and subsequently by our orthopaedic people are quite efficient. I would say is a high-class standard, okay. Err...that is my short answer saying that it is up to the standard.
			• … I would say most of, most of the existing physicians who are managing fragility fractures are well aware of secondary prevention.

	Negative perceptions	• Lack of coordination<br>• Lack of continuity of care<br>• Medical doctors lacking knowledge	• At the moment I will say everybody is dancing their own orchestra because, because … if you have a fracture, you will be under the orthopaedic team. So it is up to the orthopaedic team to refer you to the rehab team, which, if they feel that you need rehab assessment and if you need more … exercises, or for them to refer you to the endocrine team where they will start … all your medications
			• A (different departments) might not catch B, B might not catch C, C might not catch D, and then don't know what happens. The patient gets 'rojak (mixed advice).
			• Our medical officers are stationed around quite fast, one medical officer may be the first day today, I don't know who to refer [the patient] to, you (the patient) come back in six months, but … if they are senior … "I send you to rehab, you must do more exercises" … seniors tend to be … more stringent … they do more thorough check-ups compared to the newer ones who do not know anything yet. Then suddenly when they (junior doctors) refer to their seniors, "I have been doing this correctly?" "Oh no! You should do this, do this", so the first patient has already gone home without anything.
			• It's (the delivery of secondary fracture prevention) still not enough … there are some patients who come in with a broken left leg … the next time they come in with their right leg (fractured) due to a fall.
	Neutral perceptions	• Unsure of the current delivery of secondary fracture prevention<br>• Concerns about non-hip fragility fracture	• I don't have any idea … Among my friends and family members, we don't have any primary fractures so … I have no comment on secondary [fracture] prevention in our hospital.
			• I will say is very good …we have an ortho-geriatric service in our hospital, which we will review all the … hip fracture cases … but I am not too sure about the other fractures and whether [the non-hip fractures] will be captured or not.

Theme 2: Importance of secondary fracture prevention via a Fracture Liaison Service

Codes	Subcodes	Key points	Quotations
Importance of secondary fracture prevention via a FLS	Fracture begets fracture	• Risk and consequences of falls and fractures<br>• Direct and indirect burden	• It's important to prevent secondary fracture because … if we have another fracture it will cost money and people to care for the patient and it's difficult to recover and the risk of subsequent fracture is even higher, so you will lose the workforce and then you will have more medical expenses to spend on the care of the person who cannot work because of fracture.
			• I think it's very important, especially for the elderly and those who already had the primary fracture because the risk is always there.
			• I think we can see that from like a few different angles. One is from the patient's perspective, so … secondary prevention will definitely improve their overall or long-term health status, if we can reduce further fracture, subsequently, patients' functional status and also their morbidities status probably will be preserved, or at least optimised for a longer time. However, if that is not being taken care of, so their quality of life will drop quite significantly if there are multiple fractures subsequently. Then on the other hand, if we look at the patient, the healthcare setting … secondary prevention is something, we need to look at if we want to reduce, or at least improve the healthcare system … every time patients fractured and then need to be hospitalised and also undergo all these surgeries, they are taking up quite a lot of resources … some of the healthcare workers don't look at what are the cause involved … in overall … but … if one patient … having 2 or 3 fractures and needs to be fixed, so all these are accumulated, actually contribute quite a lot of … overall expenses we have to put …. we have to spend in the public health setting.
			• It is definitely important because it is a known fact that there is a risk of an elderly falling once within the year … the second one in the same year is high. And the more they get admitted, the more they get bedridden, the more surgeries they go through, they tend to deteriorate further, and the one-year mortality is high without surgery, and we found that even with surgery also, it's not like getting it down to zero. So the problem especially lower limb fracture, is that we want the prevention of immobility or the bedridden part because of all the complications … muscle wasting … pressure sore, deep vein thrombosis muscle wasting dry, stiffness … urine tract infections, depression.

	Need for assessments and treatments	• Need for bone health assessments<br>• Need for osteoporosis treatments	• After their first fracture we definitely need to either check their bone density, or check their calcium level … we help them to strengthen their bones, through medications, exercise, or through … whichever method we have, and to prevent another fracture.
	Competing priorities	• Balance the need	• The need is there, I'm sure the need is there. It's just that we need to balance the need ... is urgent or we can wait … but based on the current situation, probably we can wait for a while. But it's a good thing to have this because it will help you the patient and also the long-term, it would help to reduce the cost of treating that patient.
	Improved management of secondary fracture prevention via a FLS	• Better coordination between departments<br>• Reduce re-fracture rates<br>• Reduce mortality<br>• Enhance quality of life<br>• Providing education<br>• Improve hospital reputation	• The benefits … would be many … there will be a group of people who benefit from having a fracture liaison service. It would potentially reduce healthcare costs, would also potentially reduce the need to utilise acute inpatient services and … it can serve as a form of awareness or education, it can help to spread awareness of the importance of bone health even to people who are less involved in fracture or acute fracture care.<br>• It will help all these three fall teams to … sync the patient, so … the patient doesn't have like geriatric clinic, falls clinic, then go down to orthopaedic, another doctor tells me something else or either another person tells me something else, then goes to endocrine, another person tells me something else … having like a few clinics run a different kind of show, you gonna have like many problems, so is either everyone who had a fall or everyone who had a fracture or everyone prone to have a fracture to go to just one clinic … this person just liaison from that one area instead of geriatrician tells one thing, rehabs team tells another thing, orthopaedic tells another thing, endocrine tells another thing.<br>• It will be a systematic way of assessing patients, you will have a systematic way of asking for certain drugs that are not available in the hospital to make it available for the management of risk for fracture and make the policy of prescribing, someone would have to come out with a certain policy of prescribing certain medicine and then recruit them and manage the patient and monitor them at the same time, follow-up for maybe 10 years, 20 years. At the same time, we can prevent any potential fracture. So if we do it in a service, in a systematic manner, from, starting from screening, to treating, to policy, to

			making drug available, to prescribing rights until the end monitoring at the same time follow-up, it will be a very good approach.<br>• The incidence of refracture would be reduced and [prevent] other complications from happening … once … another fracture is prevented … it will be a good thing for the family members of the patients as well, to lessen the burden of caring for a fracture patient. I think that reduces family members, and the carer's burden.<br>• … it (FLS) will be a one-stop centre for patients to seek information … for the doctor to treat the [fragility fracture] patients … a good way of centralising the service of … risk assessment, fall prevention and fracture prevention.<br>• Education is very important … increase the awareness of the risk of fractures ... what is the prevention for osteoporosis, and then what is the prevention for falls, and how to assess whether you are prone to falls or not … these are the things that we need to educate the public and at the same time educate them to seek help if there is a need.<br>• Being a teaching hospital, you must have a special kind of service. It also would boost the image of our hospital.

Theme 3: Fracture Liaison Service Sustainability

Codes	Subcodes	Key points	Quotations
Fracture Liaison Service sustainability	Cost-effectiveness	• Statistics to support FLS implementation	• Yes, cost-effectiveness, by treating it early, and managing it early, you prevent subsequent medical burden and loss of work because of sick leave and hospitalisation … pharmacoeconomic is a very good way for you to look at, gather the information, to gather support and to kick start the service and to get the support from the top management.
			• … cost-effectiveness is important. If you can run the service at a minimal cost, without causing a spike in the current usage of the unit, and that provides benefits … many people will tend to follow. But if it is a very costly thing to do, no matter how effective it is, the moment the stakeholder has financial constrain, he will definitely cut down this service.
			• … not sure how much cost is needed, but … if you reduce admissions, you reduce the ... cost of treatment, sure that is more we can save compared to what we have to put in the cost for this service.
			• I think the most important thing is to convince the policymaker … with the evidence … the fracture reduction is reduced ... how much we can save money.
			• Cost-effectiveness is the keyword.
			• It has to have a database of all the patients with a fracture risk … part of the function of FLS is to set up the framework for the FLS.
	Support from relevant stakeholders needed	• Multidisciplinary team<br>• Incorporate into routine settings<br>• Trust issues	• A multidisciplinary team is needed … (like the) physiotherapist or occupational therapist … social workers to seek financing for patients … nurses to give appropriate nursing plans … Pharmacists to review medications of patients, and last but not least, the clinicians.
			• Getting them (FLS stakeholders) to actually put it into writing, their experiences and their good feedback, I think this would be a good way to promote the service, not just in the towns but in rural areas that can access Fracture Liaison Service.
			• … the state of our fracture liaison service here is still very much a champion-driven process … we are not there yet, we have not established it such that it does not depend on an individual champion or individual physician.
			• If you want the service to be sustainable, always recruit a new team. You can have a specialist from the existing team who is interested in taking up the

			responsibility as a chair team. But you always need a new team of human resources from your admin staff to your operator to your medical officer to your consultant to your service provider and then your networking. It has to be sustainable form, not today you have somebody, tomorrow you don't have … when you get the support from the top management, you will get the service in place, and you will need to sustain with the manpower, and your type of service has to solid enough to sustain.
			• FLS is not yet to be recognised in Malaysia … we have to gain a lot of healthcare professionals' trust in implementing this.
	Awareness regarding Fracture Liaison Service	• Lack of awareness	• I think maybe training … more awareness towards this service, and also since awareness is there, whether people (healthcare professionals) are interested to be a part of it and then … train them … they will be able to take up the service … there will be more people in the service … to carry it out.
			• It's the first time that I have heard this (FLS), to be honest, but as I read it, I see it's been implemented in other countries, in the UK first I think 1990's if not mistaken.
			• … it is the mindset of the (orthopaedic) doctors … They are interested in fixing a fracture, not doing much to prevent future fractures, so changing the mindset of a person is the one I find more difficult. We need to start educating the doctors, who are not aware of the importance of this tsunami of fractures, its importance is not given as great as the other fields of orthopaedics.
	Need for a Fracture Liaison Service coordinator	• Key person to ensure service sustainability	• You will need someone who is constantly pushing around … if not I don't think it (FLS) will continue.
			• You need to have a person dedicated to follow-up with the patient… and at the same time liaising with the doctor multidisciplinary from the clinic physicians, occupational therapist, pharmacist, nurses and physiotherapists for the patient to get support from all these healthcare professionals. So there's a need to be a middle person that needs to be there to have all the data available, at the same time follow-up action and make the clinic available for the patient to come. We don't want patients to come [to the hospital] but in the end no clinic.
			• Most definitely (need for FLS coordinators) because ownership of that service needs to come from someone constant. I believe the coordinator is the most constant … at least two that can help to alternate their duties.

		• A new post should ideally be created<br>• Job rellocation	• … if we have a specialist nurse, or … a full-time registrar … who could be trained for this purpose, we could create a post for this person … unfortunately, we are in a sub-ideal situation … getting extra staff is definitely very challenging at the current time ... So, rearranging personnel… and prioritising will be the way to go.
		• Mixed opinions regarding the candidate for FLS coordinator	• Everyone (can be a FLS coordinator), as long as is dedicated, and responsible.
			• … (hospital) nurses know how to obtain an appointment… and cost… They know how the electronic medical records work … nurses can do patient education. I think overseas, nurses play the main role in terms of patient education and counselling. But we just have to empower the nurses, train the nurses.
			• I think it's possible (for a nurse to be a FLS coordinator), but we are used to receiving (instructions from doctors), we are not used to making our own decisions. If I become the coordinator, maybe others may not be confident (with my suggestions).
			• Yes (becoming a FLS coordinator) … with a briefing of what he/she (the nurse) needs to do before starting the job … maybe with (working) experience … at least 3 to 5 years …"
			• the nurse will ask the prospects of FLS training, and why he/she should do more if there is no salary increment … and if you can provide accreditation (as a FLS nurse), and you provide a career pathway … there should be some incentives.
			• I think a pharmacist is more suited for being this liaison person because we know the medication, and we can also check on adherence right? Whether the patient is actually taking the medication or not, I think the pharmacist does play an important role.
			• I don't have any problems with (pharmacists as FLS coordinators), provided that there is enough manpower.
	Need for national policy	• Having national guidelines<br>• Guideline as a reference but need to be practical	• If you can make it into a national guideline then it will have a very big impact on the sustainability of this service and implementation in all the Ministry of Health facilities, so you benefit the whole Malaysia population rather than one institute.
			• Yes, Clinical Practice Guideline (CPG) is very important. I think CPG didn't mention much about Fracture Liaison Services, probably can improvise on that,

			add on this Fracture Liaison Services, and involve the multi-disciplinary approach.
			• The CPG can be used as a guide, but is not to be strictly followed, it also depends on what the hospital has, and what the hospital keeps and how much they can cope with, the budget that they are being given.
		• Prescribing rights by primary care physicians	• They (primary care physicians at government health clinics) cannot prescribe any bone treatment at all … as much as I want to involve them, there need to be some changes in this first … they are ideal for the continuation of care, but I am hoping that this (change in prescribing rights) is done over the subsequent few years.
