# Supplementary material for: Views of healthcare professionals regarding barriers and facilitators for a Fracture Liaison Service in Malaysia
Source: PLoS One. 2024 Jul 26;19(7):e0307919. doi: 10.1371/journal.pone.0307919 (PMC11280531; doi:10.1371/journal.pone.0307919)
Supplement: Attachment — Submitted filename: Response to reviewers_170624.docx [file pone.0307919.s004.docx]

Dear Editor,

Re: Re-submission of the manuscript: Views of healthcare professionals regarding barriers and facilitators for a Fracture Liaison Service in Malaysia

We, the authorship group, would like to thank the editor and the peer reviewers for their comments which were helpful and constructive.

We have listed our responses to the comments raised.

Reviewer #1:

1. In the “method” section, lines 95 to 101, I suggest first explaining each construct of the theory properly and then determining each equivalent in your study. You explained the equivalents of two of them and the definition of one. Explain all in both ways completely so the reader can understand the whole concept and assign each part to the assessed equivalents in your study.

Response:

We have modified the paragraph accordingly. [Methods, Page 4, Lines 90-99]

“Attitude refers to an individual’s positive or negative evaluation of performing a behaviour. In our study, we assessed stakeholders' attitudes by exploring their opinions on the current delivery of secondary fracture prevention and the perceived importance of implementing an FLS. Subjective norm involves the perceived social pressure to perform or not perform a behaviour. We investigated this construct by understanding stakeholders' perceptions of whether significant others (such as family, friends, and colleagues) support or oppose the implementation of the FLS model. Perceived behavioural control is the perceived ease or difficulty of performing a behaviour, reflecting past experiences and anticipated obstacles. We identified this by examining the barriers and facilitators stakeholders perceive in implementing and sustaining the FLS model. These constructs together shape stakeholders' intentions, indicating their willingness and readiness to implement and sustain the FLS model.”
